# Atypical Resting State Functional Neural Network in Children With Autism Spectrum Disorder: Graph Theory Approach

**DOI:** 10.3389/fpsyt.2021.790234

**Published:** 2021-12-14

**Authors:** Daiki Soma, Tetsu Hirosawa, Chiaki Hasegawa, Kyung-min An, Masafumi Kameya, Shoryoku Hino, Yuko Yoshimura, Sou Nobukawa, Sumie Iwasaki, Sanae Tanaka, Ken Yaoi, Masuhiko Sano, Yuka Shiota, Nobushige Naito, Mitsuru Kikuchi

**Affiliations:** ^1^Department of Psychiatry and Neurobiology, Graduate School of Medical Science, Kanazawa University, Kanazawa, Japan; ^2^Research Center for Child Mental Development, Kanazawa University, Kanazawa, Japan; ^3^Department of Neuropsychiatry, Ishikawa Prefectural Takamatsu Hospital, Kahoku, Japan; ^4^Faculty of Education, Institute of Human and Social Sciences, Kanazawa University, Kanazawa, Japan; ^5^Department of Computer Science, Chiba Institute of Technology, Narashino, Japan

**Keywords:** autism, MEG, graph theory, small-worldness, social communication

## Abstract

Measuring whole brain networks is a promising approach to extract features of autism spectrum disorder (ASD), a brain disorder of widespread regions. Objectives of this study were to evaluate properties of resting-state functional brain networks in children with and without ASD and to evaluate their relation with social impairment severity. Magnetoencephalographic (MEG) data were recorded for 21 children with ASD (7 girls, 60–89 months old) and for 25 typically developing (TD) control children (10 girls, 60–91 months old) in a resting state while gazing at a fixation cross. After signal sources were localized onto the Desikan–Killiany brain atlas, statistical relations between localized activities were found and evaluated in terms of the phase lag index. After brain networks were constructed and after matching with intelligence using a coarsened exact matching algorithm, ASD and TD graph theoretical measures were compared. We measured autism symptoms severity using the Social Responsiveness Scale and investigated its relation with altered small-worldness using linear regression models. Children with ASD were found to have significantly lower small-worldness in the beta band (*p* = 0.007) than TD children had. Lower small-worldness in the beta band of children with ASD was associated with higher Social Responsiveness Scale total *t*-scores (*p* = 0.047). Significant relations were also inferred for the Social Awareness (*p* = 0.008) and Social Cognition (*p* = 0.015) sub-scales. Results obtained using graph theory demonstrate a difference between children with and without ASD in MEG-derived resting-state functional brain networks, and the relation of that difference with social impairment. Combining graph theory and MEG might be a promising approach to establish a biological marker for ASD.

## Introduction

The etiology of autism spectrum disorder (ASD), a neurodevelopmental disorder characterized by stereotypic or repetitive behaviors with impaired social cognition and communication disorders (1), remains largely unknown. However, impaired social cognition and communication can be improved by early and appropriate interventions (2, 3). Its early diagnosis is nevertheless difficult in many cases because no biological marker has been established (4, 5). Consequently, clinicians must rely on symptoms.

In the field of brain imaging, researchers have characterized the functions of individual brain regions affected by ASD. However, results of more recent studies have suggested that ASD is a dysfunction of coordination over widely distributed brain regions (5–7). Assessing relations between brain regions (i.e., brain connectivity) and functions in each region can be an effective approach to extracting differences between individuals with ASD and typically developing (TD) individuals. Brain connectivity is a multi-faceted concept. In the human brain, neurons and neural populations do not function individually. They interact with other elements in a coordinated manner through their afferent and efferent connections (8). In this context, anatomical relations through bundles of axons are designated as structural connectivity, which can be measured using structural brain imaging such as MRI and diffusion tensor imaging (DTI). Structural connectivity describes the architecture of interregional connections, but it provides less information related to how neurophysiological functions are supported by this architecture. In contrast, functional connectivity is based on statistical dependencies between time series of cerebral activity in different brain regions. The connectivity can be measured using fMRI, SPECT, EEG or magnetoencephalography (MEG) (9). As such, functional connectivity is thought to form a physiological basis of information processing (10). It can therefore be of more interest. These two types of connectivity are fundamentally different. Results from earlier studies are somewhat inconsistent, but many reports of the literature describing connectivity in ASD yield a promising hypothesis: ASD is a disorder of long-range under-connectivity combined with local over-connectivity (11). However, in light of the brain's inherent complexity, any hypothesis based on such a simple measure (i.e., mean of the strength of connectivity) might be an oversimplification. To meet this challenge and to describe the properties of complex networks on a large scale, the field of neuroscience has provided graph theory (12).

Within the graph theory framework, a complex system is described as a set of relations among discrete objects. A key concept of graph theory is reduction of a complex system to a “graph”: a set of nodes (i.e., objects) and edges (i.e., relations). Using graph theory, one can describe properties of such graphs using the same parameters, irrespective of their constituent elements (10, 13). In this context, a brain network is definable as a graph in which the nodes represent brain regions and the edges linking pairs of nodes represent functional connectivity between pairs of corresponding brain regions. Based on such a graph, graph metrics represent the characteristics of the entire brain network as single numerical values. It is noteworthy that graph metrics differ from traditional measures of connectivity (e.g., means of connectivity) in two meaningful ways. First, graph theoretical analysis is applied to “graphs,” for which spatial arrangements of nodes were not considered. Particularly, edges are not weighted by the length of connections. Angles between edges are not specified. For that reason, graph metrics theoretically preserve very little or no spatial information. The results therefore might not be reconciled with earlier findings obtained for the connection-length-dependent over-connectivity and under-connectivity. Second, using graph theory, one can describe a certain property of the entire brain network in a single measure. In this sense, one need not address the difficulty posed by multiple comparisons. In contrast, for example, comparing the means of functional connectivities for each pair of brain regions can be expected to result in multiple comparisons. Erroneous inferences become more likely. With the help of graph theory, properties of a given graph can be described using various measures. Well-established and widely used measures include the mean clustering coefficient (CC), average shortest path length (cPL), and small-worldness (SW). Because CC represents the degree to which connected nodes are clustered, it is thought to represent the tendencies of the brain to process information locally (i.e., local functional segregation) (14). Characteristic path length (cPL) represents the average number of edges in shortest paths (i.e., sequence of edges connecting one node to another), where the average is taken over all possible pairs of nodes. For that reason, cPL is thought to indicate integration of information from remote brain regions (i.e., global functional integration) (14). Networks with high CC and low cPL are well-connected both locally and globally, and are therefore designated as small-world networks. Such networks are thought to represent an optimal balance between functional local segregation and global integration (15, 16). To describe how much a given network possesses small-world properties, small-worldness (SW) is defined as the ratio of normalized CC and cPL. SW is a particularly interesting property in that the healthy human brain is a small-world network (10, 17), but brain networks reportedly deviate from small-world networks in some neurological conditions such as Alzheimer's disease (17), depression (18), and schizophrenia (19).

Several studies have applied graph theory to compare the brain networks of typically developing individuals to those of patients with ASD in terms of CC, cPL, or SW. Two studies particularly examined graphs of structural connectivity (i.e., DTI). Others have emphasized functional connectivity. Among those latter studies, two used resting-state fMRI. All others have been EEG/MEG studies. Unsurprisingly considering methodological differences such as imaging modalities, participant characteristics, and graph theoretical measures, the results obtained from DTI-derived or resting-state fMRI-derived networks are inconsistent. The first report of structural networks was a study by Rudie et al. (20). They investigated structural networks generated based on DTI-derived fiber tracts in adolescents with and without ASD. Using six graph theoretical measures (i.e., CC, cPL, normalized CC, normalized cPL, SW, and modularity), the authors reported that the ASD group showed significantly lower modularity, but no significant differences were found in the other five measures. In a study conducted later by Qin et al. (21), children with ASD (2.89 ± 0.97 years old) and TD children (3.15 ± 1.12 years old) were recruited. Among seven measures (i.e., CC, cPL, normalized CC, normalized cPL, SW, global and local efficiency), their DTI-derived structural network showed significantly lower cPL and higher global/local efficiency in children with ASD. Combining those results, the structural brain network of ASD would show lower cPL and higher global/local efficiency in early childhood, after which those characteristics become less evident in later developmental stages. Adolescents with ASD would then show lower modularity than typically developing individuals. Among studies assessing functional connectivity, two used resting-state fMRI (20, 22). For one of those studies (20), Rudie et al. also investigated functional networks generated from resting-state fMRI. Results showed significantly lower CC, cPL, and modularity in the adolescent ASD group. It is noteworthy that Kaku et al. reported contradictory results: children with severe ASD were found to have significantly higher normalized CC and small-worldness than children with mild or moderate ASD (22). It remains unclear whether the relation between autistic symptoms and CC derived from resting-state fMRI is somewhat non-linear (i.e., individuals with ASD show lower CC than TD individuals, but those who have severe ASD shows paradoxically higher CC) or the relation is age-dependent (i.e., higher CC in childhood, but lower CC in adolescents corresponds to lower social communication).

All other reported graph theoretical studies of functional connectivity have used MEG or EEG. One must be cautious when comparing results of these studies because of the different imaging modalities (EEG vs. MEG), participant characteristics, and graph theoretical measures. Moreover, in MEG/EEG studies, synchronization measures differ among studies. Within the framework of those limitations, however, a consensus seems to hold that brain networks of adults with ASD constructed based on resting-state MEG/EEG derived functional connectivity show lower CC and higher cPL than those of healthy controls. However, the results are inconsistent for the younger population. The first report was of a study conducted by Pollonini et al. (23). They recruited young adults with and without ASD and investigated the functional networks generated from resting-state MEG recordings. Based on their findings, they reported significantly lower CC and higher cPL (in the broadband signal) in the ASD group than in healthy controls. From a later study, adults with ASD were also reported as having significantly lower CC (in the theta combined with the alpha band, and beta band) and higher cPL (in the beta band) than healthy controls had (24). Furthermore, Han et al. reported that children (6–11 years old) with ASD showed lower CC and lower SW in widely various frequencies, but the difference was less evident in younger children (3–6 years old) (25). It is noteworthy that the graph theoretical measures were correlated with ASD symptom severity in the alpha band. Regrettably, however, the results obtained from this study should be compared with caution because they did not exclude effects of medication. All the reports described above indicate lower CC in ASD patients than in healthy control participants. One notable exception is a study conducted by Ye et al. (26). They reported higher CC and lower cPL in the theta band for adolescents (12–15 years old) with ASD compared to healthy controls. It remains unclear whether the properties of resting state MEG/EEG-derived functional brain networks are specifically different in adolescents with ASD from those in adults or children with ASD, in that lower CC and lower SW in childhood ASD reported by Han et al. might arise as an effect of medication.

Results from studies that used non-resting state EEG/MEG are inconsistent. On the one hand, Boersma et al. investigated non-resting state EEG data (i.e., children passively viewed pictures of cars and faces) obtained from children with and without ASD (2–5 years old). Based on their findings, they reported that children with ASD had lower CC, lower SW (in theta and alpha bands), and higher cPL (in broad band) than those found for healthy control participants (27). On the other hand, Takahashi et al. investigated MEG data obtained when children actively watched animated video programs. They reported that children with ASD (4–7 years old) showed significantly higher SW in the gamma band and lower SW in the delta band, but differences in CC or cPL were not significant (28). It is noteworthy that among the three graph theoretical studies of children with ASD (25, 27, 28), lower SW has been almost consistently reported. For the other graph metrics, however, the findings are inconsistent. Considering the recording conditions [resting-state (25) vs. during visual stimulation (27, 28)], the difference might arise from atypical functional connectivity during visual information processing in ASD (29, 30), but the possible effect of medication in the study by Han et al. makes it difficult to compare the results directly. Furthermore, no earlier studies have investigated the relation between the graph metrics and ASD symptom severity after controlling for medication effects. In this context, resting-state MEG/EEG studies of children with and without ASD, excluding medication effects, might have a great merit, yet no such studies have been conducted. For such a study, it would be desirable to investigate relations between graph theoretical measures and autistic symptoms.

Therefore, for this study, we examined the resting-state MEG-derived functional network in children with and without ASD using graph theory. Furthermore, we examined the relation between graph theoretical measures and social communication. Based on results of earlier studies, we hypothesized that children with ASD show lower SW in resting-state MEG. Particularly, our hypotheses were the following: (1) Children with ASD show lower SW than TD children do. (2) Lower SW corresponds to severe ASD symptoms.

## Materials and Methods

### Experimental Design

For child participants with or without ASD, we assessed autism symptom severity using the Social Responsiveness Scale (SRS) (31). Intelligence was assessed using the Kaufman Assessment Battery for Children (K-ABC, Japanese version) (32). We recorded resting state MEG data. Signal sources are mapped onto the Desikan–Killiany atlas of 68 brain regions. Then MEG-derived functional brain networks were constructed in terms of the phase lag index (PLI). Applying graph theory, we calculated SW from the graphs of functional networks. Furthermore, as an exploratory analysis, we calculated CC and cPL for completeness. We then compared the graph metrics between ASD and TD. If a significant group effect was found in any graph metric, we investigated the relation between autism symptom severity and the graph metric.

Ideally, to calculate the necessary sample size to test our hypothesis, we had to know the effect size of having ASD on SW in exactly the same setting (e.g., using resting-state MEG and graphs of PLI-derived functional networks for children with and without ASD). However, no such earlier study has been described, as explained in the *Introduction*. We therefore expanded the search scope to include studies in which children with and without ASD were compared in terms of SW obtained from functional connectivity graphs (25, 27, 28). Unfortunately, we were unable to extract or calculate the standard effect size from those studies because of a lack of information. Consequently, we chose to assume the effect size as large because the three studies found significant effects of ASD on SW in small sample sizes: Boersma et al. compared 12 children with ASD and 19 TD children (27); Han et al. compared 20 children with ASD and 40 TD children (25); and Takahashi et al. compared 24 children with ASD and 24 TD children (28). To assess differences between two independent means in two groups, we set the effect size as 0.8, set the alpha value as 0.05, and set the (1—beta) value as 0.8. The statistical power was calculated for the one-tailed not two-tailed *t*-test because we hypothesized that children with ASD show lower SW than TD children do. The required sample size was therefore calculated as 21 for each group. For the sample size calculation, we used software (G*Power ver. 3.121.6; Heinrich-Heine-Universität Düsseldorf).

### Participants

The clinical group included 21 children with ASD recruited from Kanazawa University and affiliated hospitals. The control group included 25 TD children with no reported behavioral or language difficulty. The ASD diagnosis was made according to the Diagnostic and Statistical Manual of Mental Disorders (4th edition, DSM-IV) (33), using the Diagnostic Interview for Social and Communication Disorders (DISCO) (34), and the Autism Diagnostic Observation Schedule-2 (ADOS-2) (35, 36). We excluded children with (1) blindness, (2) deafness, (3) any other neuropsychiatric disorder, or (4) ongoing medication regimen. Written informed consent was obtained from parents before the children participated. The Ethics Committee of Kanazawa University Hospital approved the methods and procedures, which were conducted in accordance with the Declaration of Helsinki.

In case some were unable to complete the MEG recording, we recruited a few more participants than indicated as necessary by the sample size calculation. Unfortunately, however, one girl with ASD was excluded from analyses during MEG data preprocessing (see *Preprocessing* below). In consequence, we analyzed 20 children with ASD (14 boys, 6 girls, 60–89 months old) and 25 TD children (15 boys, 10 girls, 60–91 months old), which were fewer than the number indicated by the sample size calculation.

### Assessment of Social Autism Symptom Severity

Participant's autism symptom severity was assessed in the SRS: a 65-item rating scale that measures social communication and autistic mannerisms. A parent of each participant filled out the SRS. We used gender-normed T scores (SRS-T) and its five sub-scales: social awareness (SRS-AWA, ability to recognize social cues), social cognition (SRS-COG, interpreting social behavior), social communication (SRS-COM, reciprocal communication in social situation), social motivation (SRS-MOT, motivation to participate in social interactions), and autistic mannerism (SRS-MAN, circumscribed interests and stereotypy). Higher scores represent greater autism symptom severity.

The SRS can be completed by a parent or another adult informant. By virtue of this feature, it involves ratings of children in their natural social contexts and reflects what has been observed consistently over weeks or months of time, rather than merely reflecting results of a single clinical or laboratory observation (31). Nevertheless, one must be cautious when interpreting the SRS results because all such parent rating scales have important shortcomings, such as parent bias and limited reliability, compared to direct observation by expert clinicians.

### Assessment of Intelligence

Intelligence of the participants was assessed using the K-ABC. In K-ABC, skills for problem-solving abilities are interpreted as intelligence and are measured on the Mental Processing Scale (MPS) (37). Knowledge of facts, defined as achievement, is measured on the Achievement Scale (ACH). In this sense, K-ABC was developed to distinguish intelligence from knowledge (32, 37). Those scores are provided as age-adjusted standardized scores, normalized to have a mean of 100 and standard deviation of 15.

### MEG Recordings

MEG data were recorded using a 151-channel Superconducting Quantum Interference Device (SQUID) whole-head coaxial gradiometer MEG system for children (PQ 1151R; Yokogawa/KIT, Kanazawa, Japan) in a magnetically shielded room (Daido Steel Co., Ltd., Nagoya, Japan) installed at the MEG Center (Ricoh Co., Ltd., Kanazawa, Japan). We used a custom-made child-sized MEG system to measure brain responses in children because some difficulties arise when using conventional adult-sized MEG systems for young children. Using the child-sized MEG system ensures that sensors are positioned easily and effectively for the child's brain. Moreover, it ensures that head movements are constrained (38).

We undertook great effort to keep each child motionless during the recording. We instructed each child not to move the head or body to avoid motion artifacts. Then, one staff member escorted the child into the shielded room. The room was decorated with colorful pictures of cartoon characters, generally of a signature vehicle in a popular animation series. To encourage the child further to maintain a steady head position, the staff member stayed in the room. In this way, we were able to entertain most of the children. Additionally, they were monitored carefully using a video monitoring system. If the head position of the subject had obviously moved from its starting position, those related MEG data were excluded from further analyses.

Low pass filtered MEG data (500 Hz) were collected at a 2,000 Hz sampling rate. The MEG recordings were made in a resting state: the participant lay supine on a bed and gazed at a fixation cross mark projected onto a screen during the MEG recording. Then MEG data were recorded for 130 s for each participant. The time of MEG recording was between 11 A.M. and 3 P.M. No child showed a clear sign of drowsiness in terms of MEG waveforms. Ideally, longer recording durations are desirable. For children, especially children with ASD, however, it was difficult to keep them sitting still for long durations. The recording period had to be determined with compromise. Given that limitation, we adopted 50 s as the minimum, as we did for an earlier study (28) in which we analyzed artifact-free segments of length minimum of 50 s and found significantly different SW between children with and without ASD. We set the recording time as 130 s, slightly longer than 50 s, to secure a minimum of 50 s and to spare some time.

### MRI Recordings

A 1.5 T MRI scanner (SIGNA Explorer; GE Healthcare, USA) was used to obtain structural brain images from all participants and to compute individual head models for source analysis. The T1-weighted gradient echo and Silenz pulse sequence (TR = 435.68 ms, TE = 0.024 ms, flip angle = 7°, FOV = 220 mm, matrix size = 256 ×256 pixels, slice thickness = 1.7 mm, a total of 130 transaxial images) images were used for anatomical reference. All participant's MRI images were recorded.

### Co-registration of MEG on MRI Image

We co-registered the MEG and MRI images according to the marker locations. Four markers were recorded by the MEG and the MRI: midline frontal, vertex, and each bilateral mastoid process. For MEG, we used four coils generating a magnetic field. For MRI, we used four pieces of lipid capsule as markers because those are observed as high-intensity regions. Additionally, we showed points on mastoid processes, nasion, and the skull surface visually in MRI. About 15–25 points were shown for each participant.

### MEG Data Analysis

The MEG analyses were performed using Brainstorm (39), which is documented and freely available under the GNU general public license for download online.

### Preprocessing

The MEG data were preprocessed according to recommendations from the Organization for Human Brain Mapping (40). First, we downsampled the MEG data to 500 Hz. Second, we excluded three sensors from analyses because their signal quality was poor. Third, after applying notch filters (60, 120, and 180 Hz) to remove power-supply noise, we applied a band-pass filter (0.5–200 Hz). Fourth, we used the independent component analysis (ICA) to remove blink and cardiac artifacts. Finally, segments containing apparent motion noise or radio frequency interference were excluded from analyses after they were visually identified by an MEG expert who was blinded to the identities of participants. One girl's motion noise was excessive. For that reason, we excluded data obtained from this child from subsequent analyses. Data were segmented for continuous segments of 5 s. A minimum of 10 segments (50 s recording period) was accepted for each subject. Each epoch was band-pass filtered for commonly used frequency bands: delta (2–4 Hz), theta (4–8 Hz), alpha (8–13 Hz), beta (13–30 Hz), and gamma (30–60 Hz). This preprocessing procedure is identical to that used for our earlier study (28).

### Atlas-Guided Source Reconstruction and Segmenting

We performed signal source estimation using the participant's original anatomy. An anatomically constrained MEG approach that places an anatomical constraint on the estimated sources was used to estimate the brain signal sources (41). When the sources are estimated, each participant's recorded brain activity is assumed to lie in the cortical mantle. We coregistered MEG on participant's MRI images. A head model was computed using the overlapping spheres algorithm (42) with the default source space (a lower-resolution cortical surface representation, with 15,000 vertices). We used weighted minimum-norm estimation to estimate source orientation constraints (43). An identity matrix was used as noise covariance because no noise recording is available. Signal sources were grouped together into 68 regions represented in the Desikan–Killiany atlases (44). When we grouped sources, we used principal component analysis.

We chose Desikan–Killiany atlases considering the limit of spatial resolution of MEG as well as a balance between interpretability of the results and fineness of estimation. Graph theoretical analysis of the functional brain network fundamentally depends on the definition of nodes (i.e., brain regions). A parcellation scheme must be chosen that reasonably samples the brain regions. Results from fewer regions of interest (ROI) are expected to offer increased interpretability, but functionally and anatomically distinct regions can be regarded as a single ROI. Conversely, results from many ROIs finely represent functional connectivity patterns, but their interpretability might be difficult (45). Between these two extremes, Hallquist et al. reported in their recent literature review that researchers should divide the brain into at least 200 functional regions for fMRI studies (46). In MEG studies, however, more compromises must be made because of its low spatial resolution. In this context, Farahibozorg et al. investigated the optimal number of parcels while simultaneously minimizing the leakage between them. They concluded that the number was approximately 70 parcels, which is expected to reflect the limit of spatial resolution of MEG (47). Based on these considerations, we adopted the Desikan–Killiany brain atlas as suitable for this study.

### Phase Lag Index

To estimate functional connectivity between signal sources, we used the phase lag index (PLI). Although functional interactions can be measured by quantifying the phase relation between their time series (48), one must consider that reconstructed sources might contain spurious and artificial interactions because of field spread. In such cases, artificial synchrony might be observed between nearby signal sources (49). This kind of artificial synchrony can be removed by suppressing zero-lag synchrony and by detecting exclusively lagged interactions. One mixing-insensitive interaction metric, PLI, attenuates artificial interactions (50). Briefly, the instantaneous phase at each time point of the filtered waveform was calculated for each epoch using the Hilbert transform. Then, phase difference Δφ(*tk*)(*k* = 1,2,3, …, *N*, where *N* is the number of time points in an epoch) was calculated for each time point. The value of PLI between two signal sources in an epoch was obtained using the following definition (50).


(1) $$\begin{array}{r} PLI=|\frac{1}{N}\sum_{k=1}^{N}sign\left[\Delta \varphi \left(t_{k}\right)\right]\;| \end{array}$$

We used PLI to estimate phase synchrony between source pairs for each frequency band. The value of PLI is in the range of zero to one, inclusive. Two more synchronized sources have PLI that is closer to one. It is noteworthy that PLI does not indicate which of the two signals is leading in phase.

### Graph Construction and Graph Metrics

To describe brain characteristics, we used graph theory. A graph is a basic topographical representation of a network consisting of “nodes” and “edges.” The network used for this study comprised 68 nodes (brain regions defined by Desikan–Killiany) connected by weighted edges (calculated as PLI values). Therefore, an undirected weighted functional connectivity matrix (68 × 68) was constructed for each frequency band (i.e., delta, theta, alpha, beta, gamma) and for each epoch. We averaged the matrices of all epochs for each participant. Binary graph approaches were applied to simplify characteristics of a graph and to remove spurious connections. Because no formal consensus exists for a robust method for threshold selection, we set proportional threshold κ, the proportion of total connections retained, as 0.2 according to results reported for earlier studies (28, 50, 51) (A κ of 0.2 indicates that the strongest 20% of the connections were selected.). Furthermore, considering κ-dependency of graph metrics, we also investigated a set of κ for 0.1–0.3 with 0.02 increments. For these binary matrices, we calculated the most commonly used graph metrics: The clustering coefficient (CC), the characteristic path length (cPL), and small-worldness (SW) (15). Consequently, the graph metrics were obtained from each frequency band (i.e., delta, theta, alpha, beta, and gamma) for each set of proportional thresholds κ. To calculate them, we used the Brain Connectivity Toolbox (http://www.brain-connectivity-toolbox.net/, ver. 2019-03-03). Mathematical definitions of those metrics have been reported elsewhere in the literature (10, 52).

The number of connections between the nearest neighbors of a node as a proportion of the maximum number of possible connections, expressed as CC, represents how clustered a graph's nodes are. The presence of clusters in functional networks suggests organization of segregated neural processing (10). In addition, cPL represents the average length of the shortest path that must be traversed to go from one node to another. That value represents how rapidly a graph conveys information from one region to another and suggests the degree of integration of a graph (10). In rare cases, two nodes are disconnected; thereby, PL becomes infinite. To avoid this difficulty, we calculate only from connected nodes according to a method used for our earlier study (28). Additionally, SW was determined by the ratio of normalized CC and normalized cPL. When a graph has high CC and low cPL, the graph is more clustered but it conveys information more rapidly. Such a property is designated as SW. The property is thought to reflect an optimal balance of functional integration and segregation (52). To evaluate the SW of a graph, CC and cPL are adjusted because these metrics clearly depend on the numbers of nodes and edges of the graph. SW is therefore defined as the ratio of normalized CC and cPL. In this way, a graph of high SW is a network that is markedly more clustered than random networks (i.e., randomly generated networks for which the numbers of nodes and edges are the same), yet they have approximately the same characteristic path length (16). The SW measure captures this property in a single statistic (52). Normalized CC and cPL were obtained from a random network that is randomized by rewiring all edges five times. We produced 1000 random networks and their CC and cPL (hereinafter, CCrand and cPLrand) for each graph. Then, SW was found using the ratio of normalized CC and cPL (i.e., CC/CCrand and cPL/cPLrand). For each subject, CC, cPL, and SW were calculated on each frequency band.

### Statistical Analysis

Statistical analyses were conducted using software (Stata ver. 14.2; Stata Corp., College Station, TX, USA). We tested differences in age and scores in K-ABC and SRS between ASD and TD using Student *t*-tests (two-tailed). Sex difference was tested using chi-square tests.

The differences between ASD and TD on SW were tested using Student's *t*-test (one-tailed) for each frequency band (delta, theta, alpha, beta, and gamma). Then, to elucidate differences between ASD and TD on graph metrics further, we matched the two groups in terms of MPS in K-ABC while considering the possible effects of intelligence on functional connectivity (53). To improve the balance, we used coarsened exact matching (CEM) (54). Subsequently, we applied adjusted regression analysis. Particularly, we predict graph metrics based on the condition (ASD or TD) with CEM-weight for weighting. For the CEM algorithm, we temporarily coarsen (or categorize) each variable based on its distribution or on natural or intuitive divisions. Each participant is then assigned to one of a specified set of strata in which the participant characteristics are matched exactly on a set of coarsened variables. A weighting variable (CEM-weight) is generated to equalize the number of treated and control cases in one stratum. It is used for subsequent regression analyses (54). We used Sturge's rule as a binning algorithm (54). This report describes the degree of imbalance before and after matching by measurement of the multivariate L1 distance. The L1 distance represents how two groups are balanced in terms of matched variables (in our case, K-ABC). The L1 distance is a value between zero and one: a smaller value represents better balance. Our primary emphasis was examination of differences of SW for five frequency bands: delta, theta, alpha, beta, and gamma. Significance was inferred for *P* < 0.01 after Bonferroni correction for five comparisons was applied. We also analyzed cPL and CC for completeness, but we formulated no particular hypothesis related to those measures. Effect sizes were provided as *R*^2^. Although we primarily emphasized the graphs of the proportional threshold 0.2 for consistency with earlier studies (28, 38, 51), this procedure was applied for a set of κ over the range of 0.1–0.3 with 0.02 increments.

To investigate the relation between SW and autism symptom severity, we specifically examined the metric obtained from the graphs of threshold 0.2 as a representative for consistency with earlier studies (28, 38, 51). If a significant group effect was found for SW in any frequency band, then we applied a linear regression models to predict the SW based on the SRS-T score. In doing so, we assessed the relation between autism symptom severity and SW in such frequency bands. We analyzed effects of SRS on the SW only in children with ASD because we observed that SRS-T scores were much lower and homogeneous in TD children, thereby indicating the presence of the floor effect. Significance was inferred for *P* < 0.05, but we used appropriate correction for multiple comparisons if a significant group effect was found for more than one frequency band.

Furthermore, we investigated the relation between SW and SRS sub-scales to discern which sub-scale drives the effect. For sub-scale analysis, significance was inferred for *P* < 0.01 after Bonferroni correction was applied for five multiple comparisons: *social communication, social awareness, social cognition, social motivation*, and *autistic mannerisms* sub-scales.

In addition, considering the possibly different results derived from informant-based (i.e., SRS) and laboratory-based (ADOS-2) measures, we analyzed effects of ADOS-social interaction and communication scores on SW. Particularly, we applied a linear regression model to predict SW based on ADOS-social interaction and communication scores. Effect sizes were provided as *R*^2^.

Before applying any linear regression model, we verified that our data meet the assumptions for regression analysis before application of linear regression. Specifically, we used standard methods to verify linearity, normality, homogeneity of variance, model specifications, and influence. As a result, the assumption of homogeneity was violated for some regression models. Therefore, we used heteroscedasticity-robust standard errors (55).

## Results

One girl with ASD was unable to complete K-ABC because of severe psychomotor agitation. We found no significant differences in sex, age, or epoch number. Significant differences were found in the SRS total score, SRS sub-scale, Mental Processing Scale, and Achievement Scale. Table 1 presents the related results. Among the 20 children with ASD, module 1 was applied to one child, module 2 was applied to 17 children, and module 3 was applied to two children. Supplementary Table 1 presents the subject's sub-scores in ADOS-2.

**Table 1:** Characteristics of participants.

	**ASD**	**TD**	**χ^2^ or *t***	***P*-value**
*N*	20	25		
Sex (% Male)^†^	70	60	0.49	0.486
Age in months^‡^	73.5	69.2	−1.73	0.091
Epoch number^‡^	19.7	21.2	1.25	0.217
SRS total score^‡^	68.8	46.5	−7.57	<0.001^*^
**K-ABC scores**
MPS^‡^	99.2	114.5	3.15	0.003^*^
Achievement scale^‡^	95.3	106.9	2.41	0.020^*^

† *Chi-square test*.

‡ *Student's t-test*.

* *Statistical significance*.

*ASD, autism spectrum disorder; TD, typically developing children; K-ABC, Kaufman Assessment Battery for Children; SRS, Social Responsiveness scale; MPS, Mental Processing scale*.

### Group Differences in SW: One-Tailed *T*-tests

Our primary emphasis was to investigate differences in SW between the brain network of children with and without ASD. We first investigated the SW in each frequency band setting κ at 0.2 for consistency with earlier studies (15, 26, 35). Student's *t*-test showed that children with ASD had significantly lower SW in the beta band than TD children did [*t*(43) = 2.67, *p* = 0.005 for a one-tailed *t*-test and *p* = 0.011 for a two-tailed *t*-test]. The differences were not significant for any other frequency band.

**Figure 1:**
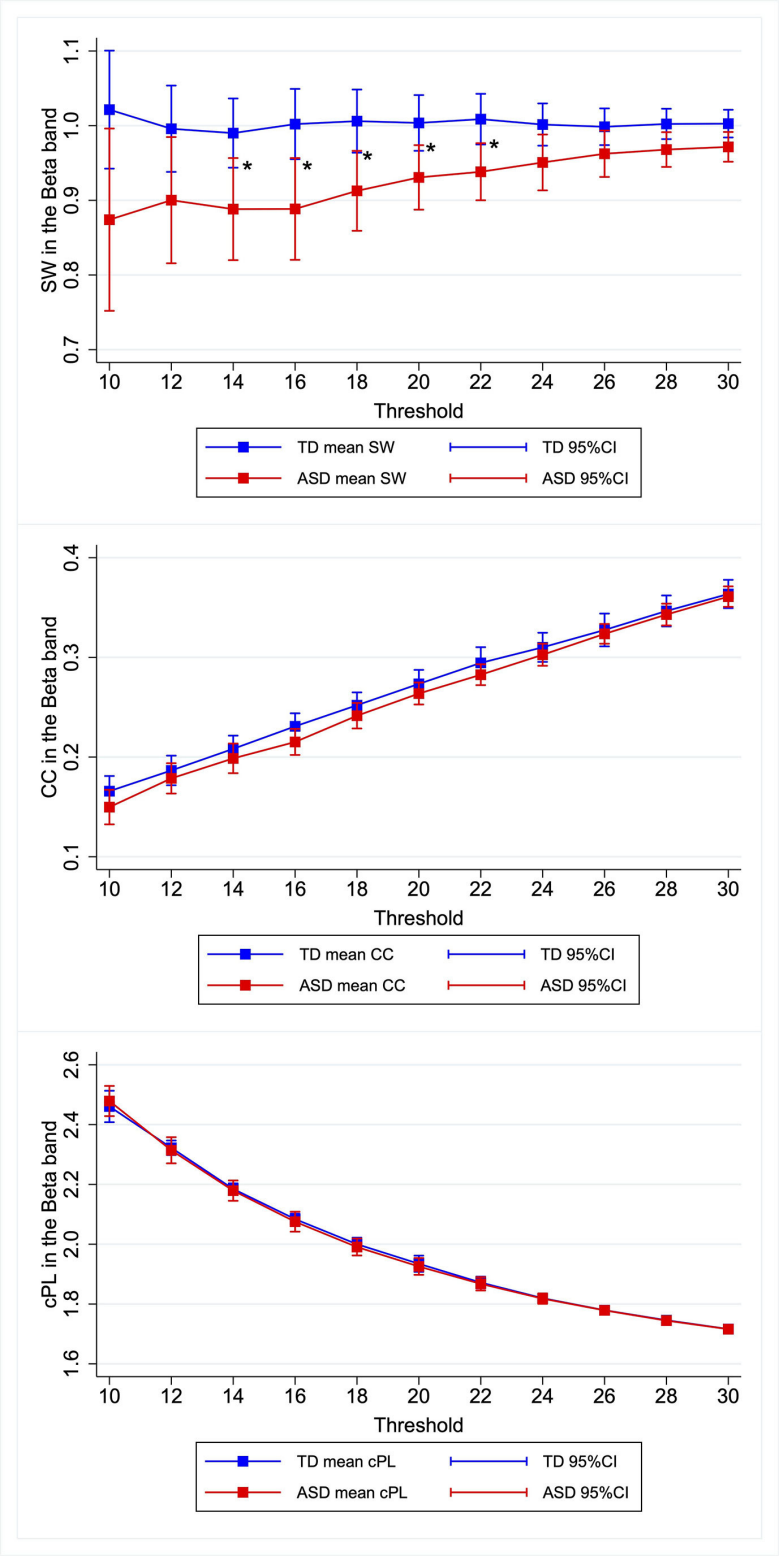
Group differences in graph metrics for different proportional thresholds in the beta band. Means of the respective graph metrics are presented with 95% confidence intervals for the respective proportional thresholds. Children with ASD show lower SW in the beta band for widely various proportional thresholds. ASD, children with autism spectrum disorder; TD, typically developing children; SW, small-worldness CC, clustering coefficient; cPL, characteristic path lengths. *Indicate statistical significance.

Similar patterns were observed for the other κ. Children with ASD showed lower SW in the beta band, the differences of which were most marked when κ was set as 0.14–0.22. Figure 1, Supplementary Figures 1–4, Supplementary Table 2 present the relevant results.

### Group Differences in Graph Metrics in Matched Participants – CEM

To investigate differences between ASD and TD on graph metrics further, we matched the two groups in terms of MPS in K-ABC considering possible effects of intelligence on functional connectivity. We first investigated the graph metrics setting κ at 0.2. After improving balance using the CEM algorithm, 15 ASD children and 25 TD children comprised the matched participants. The L1 distance improved from 0.300 to 0.115. After matching, we used linear regression with CEM weights to predict the graph metrics based on the condition (i.e., ASD or TD). Only for the model predicting SW in the beta band was the main effect of the condition (*t*(40) = −2.83, *p* = 0.007) found to be significant. Table 2 presents results obtained from the graphs with κ of 0.2. Similar patterns are observed for the neighboring κ. The differences were marked when the proportional threshold was set as 0.16–0.22. Significant difference in SW was found in the theta band when κ was set as 0.10, but the difference was not significant when κ was set as 0.12 or higher. Therefore, we discarded this result as noise. Supplementary Table 3 presents the results. Although our primary emphasis is on SW, we investigated group differences for other graph metrics (i.e., CC and cPL) for each frequency band and each κ. Significant differences were found for CC in the delta band when κ was set as 0.10 and were found for cPL in the theta band when κ was set as 0.30, but the differences were non-significant for the other κ. Hence we discarded these results as noise. We found no significant effect of group in any of the models. Supplementary Table 3 presents the results.

**Table 2:** Difference between ASD and TD in graph metrics in matched participants with κ of 0.2.

**Frequency band**	**Graph metrics**	**Coeff**.	**S.E**.	***t***	***p***	**95% CI**			***R^**2**^***
Delta	SW	0.001	0.017	0.05	0.957	−0.034	–	0.036	<0.001
	CC	−0.002	0.006	−0.31	0.758	−0.014	–	0.010	0.003
	cPL	0.005	0.005	1.05	0.301	−0.005	–	0.016	0.028
Theta	SW	−0.016	0.021	−0.75	0.456	−0.060	–	0.027	0.015
	CC	0.006	0.009	0.73	0.473	−0.011	–	0.024	0.014
	cPL	0.014	0.009	1.62	0.113	−0.003	–	0.031	0.065
Alpha	SW	−0.023	0.025	−0.90	0.375	−0.073	–	0.028	0.021
	CC	−0.022	0.019	−1.19	0.243	−0.061	–	0.016	0.036
	cPL	−0.005	0.023	−0.21	0.833	−0.052	–	0.042	0.001
Beta	SW	−0.083	0.293	−2.83	0.007^*^	−0.142	–	−0.024	0.174
	CC	−0.005	0.010	−0.54	0.592	−0.025	–	0.015	0.008
	cPL	0.008	0.018	0.44	0.661	−0.028	–	0.043	0.005
Gamma	SW	−0.009	0.032	−0.29	0.776	−0.075	–	0.056	0.002
	CC	−0.001	0.011	−0.06	0.952	−0.022	–	0.021	<0.001
	cPL	−0.006	0.012	−0.52	0.608	−0.031	–	0.018	0.007

* *Statistical significance*.

*ASD, autism spectrum disorder; TD, typically developing children; SW, small-worldness; CC, clustering coefficient; cPL, characteristic path length*.

### Relation Between SW and Autism Symptom Severity

**Figure 2:**
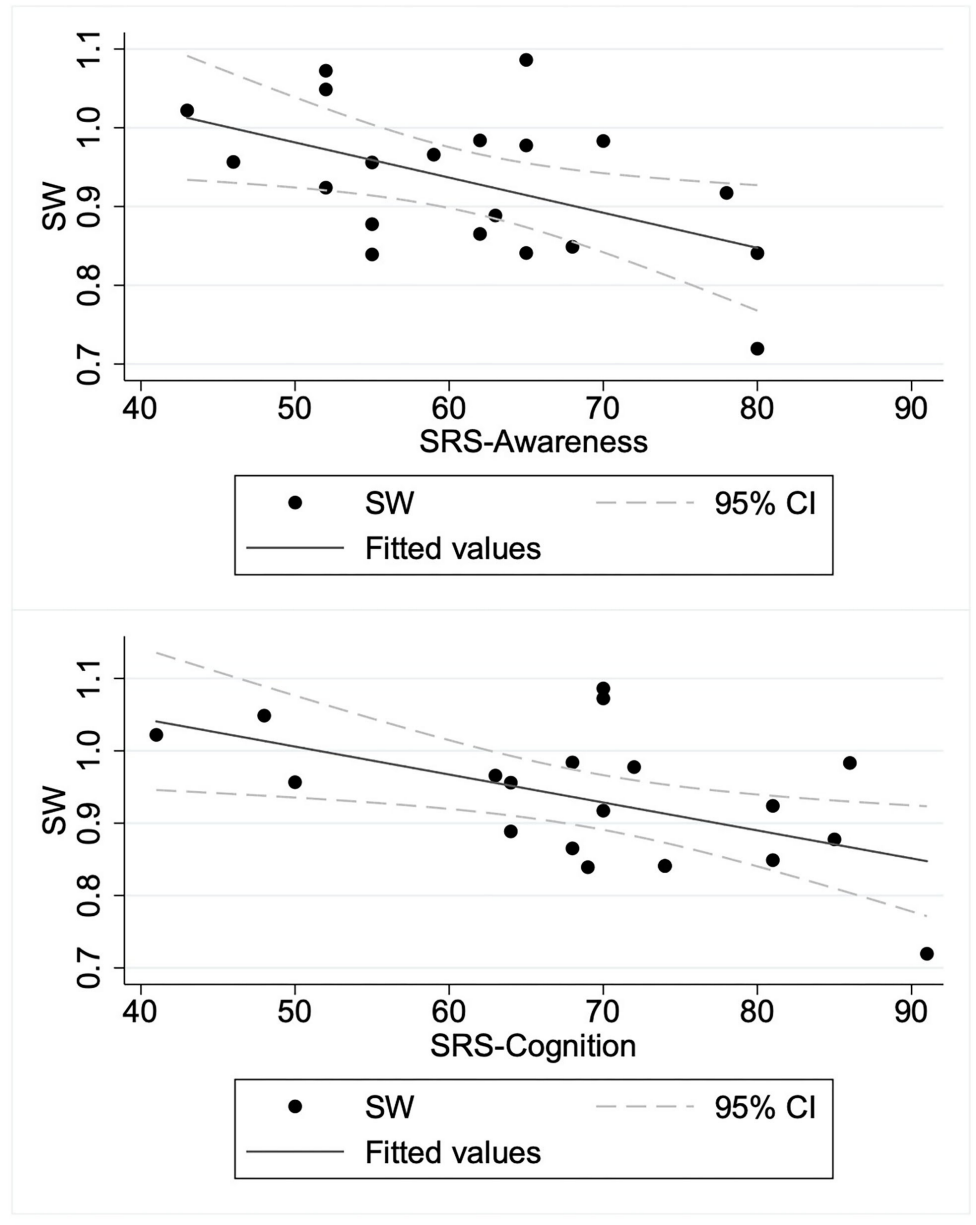
Relation between social sub-scale scores and small-worldness in children with autism spectrum disorder. ASD, children with autism spectrum disorder; SRS-Awareness, Awareness sub-scale of Social Responsiveness Scale; SRS-Cognition, Cognition sub-scale of Social Responsiveness Scale; SW, small-worldness.

To elucidate the relation between SW and autism symptom severity, we specifically examined the SW in the beta band obtained from the graphs of κ = 0.2 as a representative for consistency with earlier studies (28, 38, 51). We applied a linear regression model to predict the SW in the beta band based on SRS-T score. The regression model revealed the main effect of SRS-T score as significant (*t*(18) = −2.14, *p* = 0.047). For sub-scale analysis, a significant main effect was found only for the model predicting SW in the beta band based on the SRS-cognition sub-scale (*t*(18) = −3.00, *p* = 0.008). The higher SRS-awareness sub-scale tended to correlate with lower SW in the beta band, but the effect was non-significant after Bonferroni correction (*t*(18) = −2.69, *p* = 0.015). Those results indicate that a higher SRS-T score was associated with lower SW in the beta band in ASD children, in which SRS-cognition and SRS-awareness drove this effect. Table 3 presents the results. A graph showing the relation between social sub-scale scores and small-worldness is presented in Figure 2.

**Table 3:** Effect of SRS score on SW in the beta band in ASD participants with κ of 0.2.

**vs. SW**	**Coeff**.	**Robust S.E**.	**95% CI**			***t***	***p***	***F***	***R^**2**^***
SRS-T	−0.003	0.001	−0.006	–	0.000	−2.14	0.047^*^	4.57	0.196
SRS-AWA	−0.004	0.002	−0.008	–	−0.001	−2.69	0.015	7.23	0.258
SRS-COG	−0.004	0.001	−0.007	–	−0.001	−3.00	0.008^*^	9.01	0.281
SRS-COM	−0.003	0.002	−0.007	–	0.000	−1.81	0.088	3.27	0.154
SRS-MOT	−0.001	0.002	−0.005	–	0.003	−0.69	0.500	0.47	0.037
SRS-MAN	−0.002	0.002	−0.006	–	0.001	−1.27	0.219	1.62	0.112

* *Statistical significance*.

*ASD, autism spectrum disorder; TD, typically developing children; SW, small-worldness; SRS-T, Social Responsiveness Scale Total score; SRS-AWA, Social Awareness sub-scale; SRS-COG, Social Cognition sub-scale; SRS-COM, Social Communication sub-scale; SRS-MOT, Social Motivation sub-scale; SRS-MAN, Social Mannerism sub-scale*.

As an exploratory analysis, we applied linear regression to predict SW in the beta band based on ADOS-social interaction and communication scores. Among the 20 children with ASD, one subject was applied module 1, 17 subjects were applied module 2, and two subjects were applied module 3. Observations were therefore insufficient for module 1 and module 3. Consequently, we analyzed the effects of ADOS-social interaction and communication scores on the SW only for 17 individuals who were applied to module 2. We did not find a significant effect of ADOS-social interaction and communication scores (*t*(15) = 0.22, *p* = 0.831). This non-significant result contrasts against the significant relation found between the SRS scores and SW in the beta band. Supplementary Table 4 presents the results.

## Discussion

For TD children and children with ASD (5–8 years old), we recorded resting-state MEG data. No participant was taking medication. We constructed functional brain networks in terms of PLI and analyzed the properties of those networks to explore differences between the two groups. Children with ASD were found to have significantly lower SW in the beta band than TD children did, but not in the other frequency bands. Furthermore, in children with ASD, lower SW in the beta band corresponded to severe autistic symptoms in terms of SRS-T scores. This association was driven by the association between the SW in the beta band and SRS-cognition sub-scale. However, the relation between SW in the beta band and ADOS-social interaction and communication sub-scale was found to be non-significant.

For the beta band, we found significantly lower SW in the ASD group than in TD children. This result accords with results reported by Han et al. (25). After using resting-state EEG to investigate 6–11-year-old children, they reported that children with ASD showed lower CC and lower SW for widely various frequencies including the beta band. Nevertheless, they did not exclude effects of medication. In this context, the results obtained from the present study confirm the reduced SW in the functional brain network of children with ASD such that the difference cannot be explained by medication effects. Ye et al. similarly compared ASD and healthy control subjects, but of a slightly older age (12–15 years old) (26). In contrast to the non-significant results obtained for CC and cPL in the current study, they reported higher CC and lower cPL in the ASD group. Although comparing these findings directly is difficult because they neither explicitly exclude the effects of medication nor calculate SW, the present results can extend their results, indicating that the altered CC and cPL in the brain network of ASD would be less evident in a younger population. Boersma et al. and Takahashi et al. investigated children with and without ASD, similarly to the current study, but they used non-resting state EEG/MEG (27, 28). Particularly, Boersma et al. investigated children (aged 2–5 years) with and without ASD using EEG data obtained while children passively viewed images of cars and faces. The ASD group was found to show lower CC and higher cPL, as well as lower SW. Takahashi et al. investigated a similar age range (aged 4–7 years) using MEG data obtained while children watched animated video programs. They found that the ASD group showed higher SW in the gamma band and lower SW in the delta band, but the differences in CC or cPL were not significant. Considering atypical functional connectivity during visual information processing in ASD (29, 30), a difference between those results and the current analysis might arise from different recording conditions (i.e., resting state vs. visual stimulation). Overall, the present results provide additional evidence of age-dependent change in the resting-state functional brain networks of ASD by demonstrating that children with ASD show lower SW than those of healthy controls. Given the results from earlier studies, individuals with ASD are expected to show higher CC and lower cPL in their later developmental stage. They would then show lower CC and higher cPL during adulthood. Summarized results from earlier studies are presented in Table 4.

**Table 4:** Earlier EEG/MEG studies for ASD using graph theory.

**Authors**	**Year**	***N* (ASD vs.TD)**	**Ages**	**Device**	**Band**	**Autistic traits**
Boersma et al. (27)	2013	12 vs. 19	2–5	EEG (with Pictures)	Broad	cPL↑
					Alpha, Theta	CC↓,SW↓
Takahashi et al. (28)	2017	24 vs. 24	4–7	MEG (with Animation)	Gamma	SW↑
					Delta	SW↓
Han et al. (25)	2017	60 vs. 76	3–6	EEG	Broad	None
		20 vs. 40	6–11			CC↓, SW↓
Ye et al. (26)	2014	16 vs. 15	12–15	MEG	Theta	CC↑, cPL↓
Pollonini et al. (23)	2010	8 vs. 8	around 19^*^	MEG	Broad	CC↓, cPL↑
Barttfeld et al. (24)	2011	10 vs. 10	16–38	EEG	Beta	CC↓, cPL↑

*ASD, autism spectrum disorder; TD, typically developing children; EEG, electroencephalography; MEG, magnetoencephalography; SW, small-worldness; CC, clustering coefficient; cPL, characteristic path length*.

*↑ or ↓ indicate significant increase or decrease*.

* *Authors did not describe the age range of participants. 18.7 ± 0.7 for ASD group, 19.0 ± 1.2 for TD group*.

To date, only a report by Han et al. has described a study investigating the association between graph theoretical measures and autistic symptoms. However, they did not exclude medication effects (25). For that study, ASD symptom severity as measured by the autism behavior checklist (56) was negatively correlated with CC and positively correlated with cPL in the alpha band. This report is therefore the first describing that ASD symptom severity measured by SRS is related to a graph metric (i.e., SW in the beta band) of resting-state MEG-derived functional brain networks in childhood ASD after controlling for medication effects. This association was driven by association between the SW and SRS-cognition sub-scale. Against our expectations, however, the relation between ADOS-social interaction and communication scores and the SW was not found to be significant. This inconsistency might arise from the different results derived from parent ratings (i.e., SRS) and clinical observations (ADOS-2). Those different results might be explained by contextual factors and different perspectives. Parents might have more opportunities to observe their child's everyday behaviors. Such behaviors might not be apparent during brief one-to-one test situations (i.e., the controlled test setting for ADOS-2). However, parents are not necessarily trained to differentiate and capture autistic behaviors, whereas clinicians are trained extensively to be able to capture autistic behaviors accurately. Clinicians also have vast amounts of knowledge about typical and atypical development of children. For these reasons, by combining results from two regression analyses (SW vs. SRS scores and SW vs. ADOS-social interaction and communication score), it might be found that lower SW in children with ASD corresponds to fewer social behaviors appearing only in situations outside of an examination room. However, the results should be interpreted with caution because of major limitations: parent ratings entail parent bias and provide lower reliability than direct observations made by expert clinicians.

Some limitations must be described. First, most of the children with ASD examined for this study were high-functioning children who were able to remain stationary during MEG measurements. Therefore, the findings might not be applicable to children with lower verbal or intellectual abilities, or who have difficulty remaining stationary. Second, we computed PLI for each of >10 epochs of 5 s. This shorter epoch length might affect the PLI values and PLI-based graph metrics. For that reason, one must be cautious when comparing the present results with those from other studies using different epoch lengths. For example, Fraschini et al. analyzed effects of epoch length on PLI and PLI-derived graph metrics using six epochs for each epoch length (i.e., 1, 2, 4, 6, 8, 10, 12, 14, and 16 s) (57). They reported that, in the source space, PLI values and PLI-based graph metrics (weighted CC and weighted cPL) show a decrease with increasing epoch length, where the results stabilize for epochs with lengths of longer than 10 s. Furthermore, at the network level, shorter epochs showed less clear patterns of PLI than those obtained from longer epochs, possibly reflecting inter-epoch variation in neuronal activity. It is noteworthy, however, that their study found no significant relation between epoch length and the standard error of the mean PLI and PLI-based graph metrics with better behavior (in terms of stability) was observed for measures extracted from source level analyses compared with results obtained from sensor level analyses. Although it is difficult to compare those results directly because of methodological differences (e.g., EEG vs. MEG, frequency bands, source estimation methods), the evidence suggests that the results presented herein might be valuable in terms of test–retest reliability, but one must be cautious when comparing results from different studies using data obtained using different epoch lengths. Third, we exerted great effort to keep the participants motionless. Despite that effort, it is still possible that motion artifacts resulted from subtle movement during the acquisition. In addition, the head motion possibly differed between the two groups. Unfortunately, we did not have access to head motion data from MEG scans to ascertain whether this was true. Further study using a quantification algorithm for head movement can be expected to help clarify how motion artifacts affect the graph metrics. Fourth, comparison of the results obtained from this study with those from other graph theoretical studies using different quantities of ROIs (i.e., number of nodes) and different proportional thresholds should be done only with due caution. An important difficulty arises from the fact that graph metrics depend on the number of graph nodes and edges (58). Especially, the dependence cannot be neglected when the nodes are fewer than 200 (58). No satisfactory method for correcting for such effects has been reported in the relevant literature. Fifth, the sample was smaller than the sample size calculation indicated. A study with low statistical power has a reduced likelihood that a significant result reflects a true effect. In fact, it might overestimate the effect size (59). For that reason, to estimate the effect sizes of group difference (i.e., ASD vs. healthy controls) accurately in graph metrics, studies examining a larger sample must be conducted. Sixth, one must be cautious when interpreting the results of parent-report measures such as SRS because all such scales entail important shortcomings, such as parent bias and limited reliability, compared to direct observation by expert clinicians.

For this study, we specifically examined young TD children and children with ASD because early diagnosis of ASD is important for supporting developmental follow-up in children with ASD. Our study provides important information that can be expected to improve our understanding of neurophysiological mechanisms underlying the earlier development of social abilities and brain networks in children with ASD. As a highly non-invasive method, MEG can provide a potential biomarker for ASD in young children by application of the observed behavioral and neurophysiological alterations in patients with ASD.

## Data Availability Statement

The raw data supporting the conclusions of this article will be made available by the authors, without undue reservation.

## Ethics Statement

Written informed consent was obtained from the minor(s)' legal guardian/next of kin for the publication of any potentially identifiable images or data included in this article.

## Author Contributions

DS, TH, CH, K-mA, and MK: contributed to conception and design of the study. CH, K-mA, YY, SI, ST, and KY: acquired the data. DS and SN: wrote source code for analysis. DS, TH, and MK: analyzed the data. DS: wrote the first draft of the manuscript. TH and MK: wrote sections of the manuscript. All authors contributed to manuscript revision, read, and approved the submitted version.

## Funding

This study was supported by the Center of Innovation Program of the Japan Science and Technology Agency, JST, JSPS KAKENHI Grant Numbers: 20H03599 and 20K16623.

## Conflict of Interest

The authors declare that the research was conducted in the absence of any commercial or financial relationships that could be construed as a potential conflict of interest.

## Publisher's Note

All claims expressed in this article are solely those of the authors and do not necessarily represent those of their affiliated organizations, or those of the publisher, the editors and the reviewers. Any product that may be evaluated in this article, or claim that may be made by its manufacturer, is not guaranteed or endorsed by the publisher.
