# Supplementary material for: Atypical Resting State Functional Neural Network in Children With Autism Spectrum Disorder: Graph Theory Approach
Source: Front Psychiatry. 2021 Dec 14;12:790234. doi: 10.3389/fpsyt.2021.790234 (PMC8712628; doi:10.3389/fpsyt.2021.790234)
Supplement: Supplementary file 1 [file Data_Sheet_1.DOCX]

Supplementary Materials

# Supplementary Figures and Tables

## Supplementary Figures

**Supplementary Figures 1-4. Group differences in graph metrics for different proportional thresholds.**Means of the respective graph metrics are presented with 95% confidence intervals for the respective proportional thresholds. No significant difference was found from thresholds with the delta, theta, alpha, or gamma band.
ASD, children with autism spectrum disorder; TD, typically developing children; SW, small-worldness CC, clustering coefficient; cPL, characteristic path lengths

## Supplementary Tables

**Supplementary Table 1. Scores and subscores of ADOS-2 in children with autism spectrum disorder**

**Supplementary Table 2. Differences between ASD and TD on SW assessed using Student's *t*-test**

**Supplementary Table 3. Differences between ASD and TD in graph metrics in matched participants with the respective thresholds**

**Supplementary Table 4. Effects of ADOS2 score on SW in the beta band with κ of 0.2**
